# Evaluating the completeness of the reporting of abstracts since the publication of the CONSORT extension for abstracts: an evaluation of randomized controlled trial in ten nursing journals

**DOI:** 10.1186/s13063-023-07419-5

**Published:** 2023-06-22

**Authors:** Yan He, Rong Zhang, Wenjing Shan, Yuhuan Yin, Xiaoli Zhang, Yiyin Zhang, Xiaoping Wang

**Affiliations:** 1 Health Human Resources Service Center, Health Commission of Gansu Province, Lanzhou, Gansu 730000 China; 2 School of Nursing, Gansu University of Chinese Medicine, Lanzhou, 730000 Gansu China; 3 Oncology Department, Nursing, Xi’an International Medical Center, Xi’an, 710000 Shaanxi China; 4 Urological Examination Room, Gansu Provincial Hospital, Lanzhou, 730000 Gansu China

**Keywords:** Abstracts, Randomized controlled trials, Consolidated Standards of Reporting Trials for Abstracts

## Abstract

**Background:**

As a practice-oriented discipline, strict adherence to reporting guidelines is particularly important in randomized controlled trial (RCT) abstracts of the nursing area. However, whether abstract reports after 2010 have complied with the Consolidated Standards of Reporting Trials for Abstracts (CONSORT-A) guideline is unclear. This study aimed to evaluate whether the publication of CONSORT-A has improved abstract reporting in nursing and explores the factors associated with better adherence to the guidelines.

**Methods:**

We searched the Web of Science for 200 RCTs randomly selected from ten nursing journals. We used a data extraction form based on CONSORT-A, including 16 items, to analyze the reporting adherence to the guidelines, and the reporting rate of each item and the total score for each abstract were used to indicate adherence and overall quality score (OQS, range 0–16). A comparison of the total mean score between the two periods was made, and affecting factors were analyzed.

**Results:**

In the studies we included, 48 abstracts were published pre-CONSORT-A whereas 152 post-CONSORT-A. The overall mean score for reporting adherence to 16 items was 7.41 ± 2.78 and 9.16 ± 2.76 for pre- and post-CONSORT-A, respectively (total score: 16). The most poorly reported items are “harms (0%),” “outcomes in method (8.5%),” “randomization (25%),” and “blinding (6.5%).” Items including the year of publication, impact factor, multiple center trial, word count, and structured abstract are significantly associated with higher adherence.

**Conclusions:**

The adherence to abstract reporting in nursing literature has improved since the CONSORT-A era, but the overall completeness of RCT abstracts remained low. A joint effort by authors, editors, and journals is necessary to improve reporting quality of RCT abstracts.

## Introduction

Randomized controlled trials (RCTs) are considered the best source of evidence for clinical practice and decision-making [1]. Therefore, accurate and complete reporting of RCT results is essential for helping readers critically appraise RCT outcomes [2]. This has led to the development of standardized reporting guidelines for RCTs, such as the Consolidated Standards of Reporting Trials (CONSORT), which was established in 1996 [3] and last updated in 2010 [4].

With the publication of large volumes of RCTs, most readers initially evaluate the articles by reading their abstracts to understand how a clinical trial was conducted, so as to determine whether or not to conduct a more in-depth full-text analysis [2]. Marcelo et al.’s study showed that more than a third of doctors routinely used abstracts to answer clinical questions [5]. Therefore, an accurate summary of the study content in an abstract is essential for allowing the reader to get a good synopsis of the study. However, there is considerable evidence that the reporting quality of RCT abstracts was suboptimal [6]. Considering the importance of RCT abstracts, an extension to the CONSORT for Abstract (CONSORT-A) was published in 2008 [7], which summarizes a list of minimal items that should be reported in abstracts. The publication of CONSORT-A has been endorsed by the World Association of Medical Editors, the International Committee of Medical Journal Editors, and the Council of Science Editors [8]. However, despite the publication of the guidelines, studies in different areas and certain specialties have suggested that the reporting adherence of RCT abstracts to the guidelines remained suboptimal [5, 9–13].

With the number of RCTs published in nursing journals increasing dramatically, one question that has not been fully answered is whether the qualitative growth of research is the same as its quantitative growth. Systematic reviews determined that the reporting quality of RCTs in nursing needed improvement [14, 15]. Currently, in the nursing area, we found only one study that evaluated the reporting quality of RCT abstracts published between 1984 and 2010 [16]. However, whether abstract reports after 2010 have complied with the CONSORT-A guidelines is still being determined. As a practice-oriented discipline, strict adherence to reporting guidelines is particularly important in RCT abstracts of the nursing area. We aim to assess the adherence of abstracts to the CONSORT-A checklist in ten high-impact nursing journals and to analyze the factors associated with higher CONSORT-A scores.

## Methods

### Study design

We conducted a descriptive study based on the literature from February to April 2021. We selected ten high-impact nursing journals, evaluated the compliance of article abstracts published in these journals with the CONSORT-A, and analyzed the factors related to higher CONSORT-A scores.

### Data source and search strategy

Based on the Journal Citation Reports 2018, we selected the ten nursing journals with the higher “impact factors” in 2018: *Journal of Cardiovascular Nursing*, *International Journal of Nursing Studies*, *European Journal of Cardiovascular Nursing*, *Journal of Nursing Scholarship*, *Nurse Education Today*, *Birth-Issues in Perinatal Care*, *Women and Birth*, *Nursing Outlook*, *European Journal of Cancer Care*, and *Journal of Family Nursing*. Under the article submission instructions for the authors, the selected journals clearly recommended following the CONSORT statement, whereas the CONSORT-A was not explicitly endorsed.

We searched Web of Science to identify literature from all “randomized controlled trials” or “clinical trials” published in these journals from inception to December 31, 2020, limited to 10 selected nursing journals. If the abstract reports on RCTs of any design, the abstract is included. If the abstract of a potentially relevant article is unclear, we retrieve and evaluate the full text to see if the study reported RCTs. We did not search the grey literature, and no limitations were made on the language. The results from the database search were imported into EndNote X5. Duplicate records were identified and removed.

### Eligibility criteria

RCTs whose primary purpose was to ascertain the effectiveness of nursing interventions were included. We defined “nursing intervention” as patient care activities performed by registered nurses focused on improving health. To be considered for inclusion, the nursing interventions must have been administered without other interventions. We placed no limitations on the types of intervention, study population, or clinical setting. We included RCTs in which the allocation of participants to interventions was described by the words such as random, randomly allocated, randomized, or randomization. The exclusion criteria are as follows: RCTs that did not have an abstract, observational studies, economic analyses on RCTs, quasi-randomized trials, cluster randomized trials, diagnostic or screening tests, subgroup or secondary analyses of previously reported RCTs, and editorials, letters, or news reports. We did not consider conference abstracts because such types of publications are generally not peer-reviewed.

### Sample size calculation and study selection

The primary objective of this study is to compare the mean adherence of abstracts to the CONSORT-A checklist in the prepublication versus postpublication period based on 16 items. To determine the sample size, we used the “rough rule of thumb” advocated by Kendall et al. in which the number of samples should be at least 5 to 10 times the number of items [17]. As we have 23 items in this study, 7 for trial characteristics and 16 items from the CONSORT-A checklist, we have calculated the sample size as follows: 23 × 5 to 23 × 10, with the final sample number between 115 and 230. Finally, 200 studies were randomly selected for the final analysis.

The study selection was conducted independently by two researchers (YYH and ZXL). First, they reviewed the title and then the abstract of each citation and decided its appropriateness for inclusion. In case of doubt, the full text was downloaded to judge whether the article was indeed an RCT. Any disagreement was solved by consensus. As more than 200 records were identified, the abstracts of the potentially included records were imported into Microsoft Excel and were randomly ordered using a computer-generated sequence. The first 200 records were selected for further analysis.

### Pilot study

In calculating the interobserver agreement [18], the Cohen *k* statistic was used; the agreement was categorized as poor (≤ 0.00), slight (0.01–0.20), fair (0.21–0.40), moderate (0.40–0.60), substantial (0.61–0.80), and almost perfect (0.81–1.00). The interobserver agreement is calculated for the purpose of studying selection.

### Data extraction and evaluation

Two reviewers (YYH and YYZ) independently extracted the data related to the quality of reporting using a standardized and pilot-tested data collection form based on the CONSORT-A. The extracted abstract data items are checked consistently, and the differences are resolved through discussion. One item in CONSORT-A is related specifically to conference abstracts. As we did not include conference abstracts, this item was removed from our assessment. We refined several of the items included in the CONSORT-A checklist to assess certain items in more detail, as shown in Table 2. In addition, we also extracted the following information from each abstract for trial characteristics: year of publication (before 2009 and 2009–2018), the journal impact factor (< 3 and > 3), number of authors (< 3, 4–6, ≥ 7), center (single or multicenter), sample size (< 100 and ≥ 100), word count (≤ 250 and > 250), and abstract format (structured and unstructured). That is, our final data extraction table includes 7 trial characteristics and 16 items from the CONSORT-A checklist.

Each item was given “yes,” “unclear,” or “no” response depending on the level of reporting of each abstract. In addition, based on previous studies [5, 19–21], to calculate the adherence score of each abstract, all items were scored with equal weight. The item was scored 1 if it was well reported, 0 if it was not reported, and 0.5 if it was inadequately reported, just for the items having subtitles (at least one subtitle was adequately reported) [19]. Then, an overall quality score (OQS, range 0–16) was developed by summarizing the individual score (1/0.5/0) across all 16 items; the higher score is regarded as a better adherence.

### Data analysis

To make a comparison of the overall adherence to the CONSORT-A checklist over time, we divided time into two periods: pre-CONSORT-A publication (before 2009) and post-CONSORT-A publication (2009–2019). Variables were summarized using descriptive statistics, namely absolute (*n*) and relative (%) frequencies for categorical variables and mean ($\overline{x }$) and standard deviation (SD) for numerical variables. The Pearson chi-square test was used to analyze the reported differences of each item of CONSORT-A in two periods. We used the total CONSORT-A score to reflect the reporting quality of these RCT abstracts. Multiple linear regression analysis was performed to analyze the influencing factors of the CONSORT-A score. The dependent variable was the CONSORT-A score, and the independent variables were number of authors, number of centers, journal impact factor, sample size, structured abstract, word count, and year of publication. During the multivariable modeling, the variance inflation factor (VIF) was used to detect multicollinearity. Any predictor with a VIF above ten was excluded from the final model. Data analyses were performed by the SPSS statistical software (version 21.0), and *P* < 0.05 was treated as of statistical significance.

### Ethical considerations

Ethical approval did not apply to this study, as the study did not involve human or animal testing, and the included RCT abstracts can be obtained from databases.

## Results

**Fig. 1:**
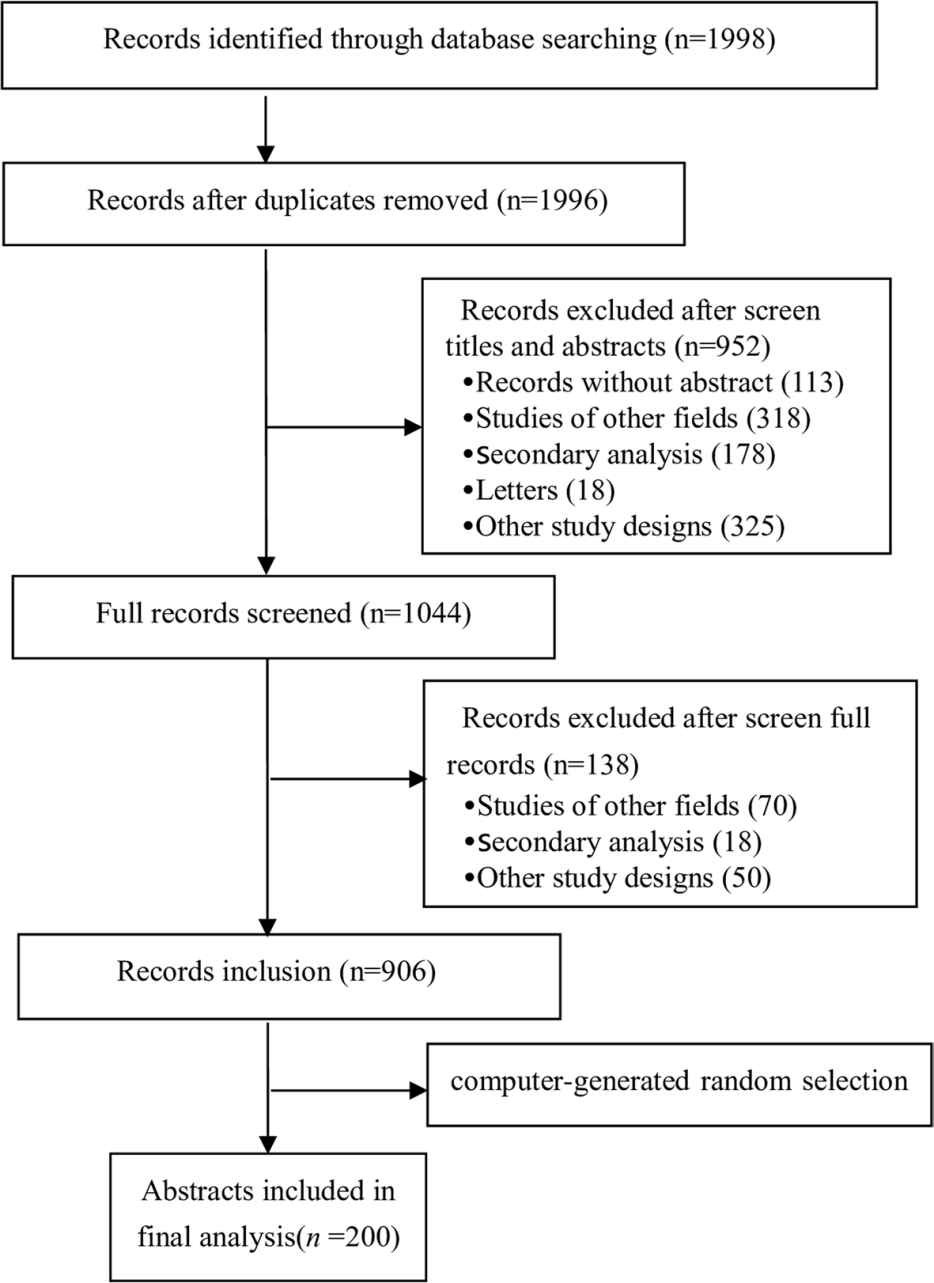
Flow chart for the study selection

Our search yielded 1996 RCTs initially; after the title and abstract screening, 906 potentially eligible articles were identified, with 190 published before 2009 and 716 published after 2009. Of them, 200 were randomly chosen for the final analysis. A detailed flow diagram of the literature search and identification of nursing RCT abstracts are depicted in Fig. 1.

### Agreement of reviewers

In the pilot study, the inter-observer concordance for article selection was 0.844, increasing to 0.913 after all disputed items were agreed upon by the third researcher. This indicates that the inter-observer reliability was almost perfect.

### Characteristics of included abstracts

The characteristics of the included abstracts were shown in Table 1. Twenty-four percent were published pre-CONSORT-A and 76% post-CONSORT-A. Nearly 80% of studies were multicenter, more than 70% were published in journals with an impact factor of less than 3, and more than 80% were structured abstracts.

**Table 1:** Trial characteristics of the included abstracts (*N* = 200)

Characteristics	Before CONSORT-A (*N* = 48), *n* (%)	After CONSORT-A (*N* = 152), *n* (%)	Overall CONSORT-A, *n* (%)
Journal			
* Journal of Cardiovascular Nursing*	3 (6.3)	13 (8.6)	16 (8.0)
* International Journal of Nursing Studies*	13 (27.1)	77 (50.7)	90 (45.0)
* European Journal of Cardiovascular Nursing*	3 (6.3)	20 (13.2)	23 (11.5)
* Journal of Nursing Scholarship*	2 (4.2)	5 (3.3)	7 (3.5)
* Nurse Education Today*	2 (4.2)	16 (10.5)	18 (9.0)
* Birth-Issues in Perinatal Care*	21 (43.8)	4 (2.6)	25 (12.5)
* Women and Birth*	0 (0.0)	7 (4.6)	7 (3.5)
* Nursing Outlook*	0 (0.0)	1 (0.7)	1 (0.5)
* European Journal of Cancer Care*	4(8.3)	6 (3.9)	10 (5.0)
* Journal of Family Nursing*	0 (0.0)	3 (2.0)	3 (1.5)
Number of authors (*n*)			
≤ 3	22 (45.8)	47 (30.9)	69 (34.5)
4–6	21 (43.8)	66 (43.4)	87 (43.5)
≥ 7	5 (10.4)	39 (25.7)	44 (22.0)
Centers			
Single center	12 (25.0)	34 (22.4)	46 (23.0)
Multicenter	36 (75.0)	118 (77.6)	154 (77.0)
Journal impact factor			
< 3	35 (72.9)	75 (49.3)	110 (55.0)
≥ 3	13 (27.1)	77 (50.7)	90 (45.0)
Sample size			
< 100	23 (47.9)	68 (44.7)	91 (45.5)
≥ 100	25 (52.1)	84 (55.3)	109 (54.5)
Structured abstract			
Yes	33 (68.8)	130 (85.5)	163 (81.50)
No	15 (31.3)	22 (14.5)	37 (18.50)
Word count			
≤ 2502	30 (62.5)	53 (34.9)	83 (41.5)
> 250	18 (37.5)	99 (65.1)	117 (58.5)

*CONSORT-A*, Consolidated Standards of Reporting for Abstract; *Before CONSORT-A*, before 2009; after CONSORT-A: 2009–2019

### Completeness of reporting for the CONSORT-A items

Table 2 showed the adherence of these RCT abstracts to the CONSORT-A checklist. Items with a reporting rate of more than 80% in both periods were interventions and numbers analyzed. Items with a reporting rate of more than 90% in both periods were the conclusions. Items with a reporting rate of less than 20% in both periods were the details of blinding, the outcomes in the “Methods” section, trial status, and harms, although the proportion of randomized details reported in abstracts after 2009 has increased compared to pre-2009, the reporting rate was still less than a third.

**Table 2:** Adherence of nursing RCT abstracts to CONSORT-A checklist

Items	Description	Before CONSORT-A (*N* = 48), *n* (%)	After CONSORT-A (*N* = 152), *n* (%)	Overall adherence, *n* (%)	*P*
1. Title	Identification of the study as randomized	28 (58.3)	114 (75.0)	142 (71.0)	0.027
2. Trial design	Description of the trial design (e.g., parallel, cluster, non-inferiority)	40 (83.3)	107 (70.4)	147 (73.5)	0.077
**Methods**					
3. Participants	Eligibility criteria for participants and the settings where the data were collected	28 (58.3)	103 (67.8)	131 (65.5)	0.231
	a. Eligibility criteria for participants	2 (4.2)	14 (9.2)	16 (8.0)	0.261
	b. Settings of data collection	5 (10.4)	23 (15.1)	28 (14.0)	0.412
4. Interventions	Interventions intended for each group	39 (81.2)	125 (82.2)	164 (82.0)	0.877
5. Objective	Specific objective or hypothesis	35 (72.9)	117 (76.9)	152 (76.0)	0.566
6. Outcome^a^	Clearly defined primary outcome for this report	2 (4.2)	15 (9.9)	17 (8.5)	0.217
7. Randomization	How participants were allocated to interventions	4 (8.3)	46 (30.3)	50 (25.0)	0.002
	a. Random assignment	28 (58.3)	100 (65.8)	128 (64.0)	0.348
	b. Sequence generation	1 (2.1)	5 (9.6)	6 (3.0)	0.669
	c. Allocation concealment	0 (0.0)	0 (0.0)	0 (0.0)	/
8. Blinding (masking)	Whether or not participants, caregivers, and those assessing the outcomes were blinded to the group assignment	2 (4.2)	11 (7.3)	13 (6.5)	0.452
	a. Generic description only (for example, single-blind, double-blind)	10 (20.8)	68 (44.7)	78 (39.0)	0.003
**Results**					
9. Numbers randomized	Number of participants randomized to each group	36 (75.0)	121 (79.6)	157 (78.5)	0.498
10. Recruitment	Trial status	5 (10.4)	28 (18.4)	33 (16.5)	0.193
11. Numbers analyzed	Number of participants analyzed in each group	40 (83.3)	127 (83.6)	167 (83.5)	0.972
	a. Intention-to-treat analysis or per-protocol analysis	1 (2.1)	11 (7.2)	12 (6.0)	0.190
12. Outcome^b^	For the primary outcome, a result for each group and the estimated effect size and its precision	11 (22.9)	78 (51.3)	89 (44.5)	0.001
	a. Primary outcome result for each group	4 (8.3)	9 (5.9)	13 (6.5)	0.555
	b. Estimated effect size	3 (6.3)	23 (15.1)	26 (13.0)	0.111
	c. Precision of the estimate (for example, 95% confidence interval)	3 (6.3)	6 (3.9)	9 (4.5)	0.502
13. Harms	Important adverse events or side effects	0 (0.0)	0 (0.0)	0 (0.0)	/
14. Conclusions	General interpretation of the results	44 (91.7)	139 (91.4)	183 (91.5)	0.962
	a. Benefits and harms balanced	0 (0.0)	0 (0.0)	0 (0.0)	/
15. Trial registration	Registration number and name of the trial register	12 (25.0)	75 (49.3)	87 (43.5)	0.003
16. Funding	Source of funding	25 (52.1)	85 (55.9)	110 (55.0)	0.899

*CONSORT-A*, Consolidated Standards of Reporting Trials for Abstracts

^a^Outcome reported in the “Methods” section

^b^Outcome reported in the “Results” section

### Assessment of overall reporting adherence to the CONSORT- A checklist items

The adherence proportion for the majority of items in the CONSORT-A checklist was presented as above 50%, and no items except the conclusion items reached 90% compliance scores. When reporting compliance for 16 items, the overall mean score before CONSORT-A was 7.41 ± 2.78 and after CONSORT-A was 9.16 ± 2.76 (total score: 16). We compared the mean difference between the two periods, and the overall mean score after CONSORT-A was higher than before CONSORT-A; RCTS published after CONSORT-A showed higher compliance to CONSORT-A, with a mean difference of − 1.75 (95% CI 0.85 to 2.65, *P* < 0.001).

### Factors associated with the overall reporting adherence

Multiple linear regression results in Table 3 showed that higher CONSORT-A scores were associated with multicenter studies (*P* < 0.001), higher journal impact factor (*P* < 0.001), structured abstracts (*P* = 0.009), more word counts (*P* < 0.001), and more recent publication (*P* = 0.032).

**Table 3:** Linear regression analysis of factors affecting the overall adherence score

Characteristics	Unstandardized coefficients		*P*	95% CI		VIF
	*B*	SE		Lower	Upper	
Number of authors						
≤ 3	Reference					
4–6	0.08	0.06	0.171	− 0.04	0.20	1.38
≥ 7	0.11	0.06	0.086	− 0.02	0.23	1.45
Number of centers (multicenter)	2.10	0.38	< 0.001	1.36	2.84	1.10
Journal impact factor (≥ 3)	2.31	0.47	< 0.001	1.37	3.24	2.45
Sample size (≥ 100)	0.04	0.49	0.943	− 0.94	1.01	2.59
Structured abstract (yes)	1.09	0.41	0.009	0.28	1.90	1.12
Word count (> 250)	1.26	0.31	< 0.001	0.65	1.87	1.05
Year of publication (2009–2018)	0.81	0.37	0.032	0.07	1.54	1.13

## Discussion

Our study found that compared to pre-2009, the average CONSORT-A score for RCT abstracts published in 10 nursing journals since 2009 increased by 1.75 points, with three items: title, outcomes (result section), and trial registration have increased by more than 20% in average reporting rate. Such an upward trend in the quality of abstract reporting over time was observed not only in the nursing field but also in other fields, such as in heart failure trials [22], oncology trials [23], COVID-19 intervention trials [24], and trials in endodontics [11]. Although with slight improvement, there is still much work needed, especially in less reported items, including important adverse events or side effects, allocation concealment, and sequence generation. Thus, clinicians who use data in the abstract to make healthcare decisions could get information as much as possible [25].

Our study showed the outcomes (“Methods” section), randomization, blinding, recruitment, and harms with a poor report rate, and among these, randomization, blinding, and harms constituted the important information to ensure the validity of trials [25]. Although the proportion of randomized details reported in abstracts after 2009 has increased compared to pre-2009, the reporting rate was still less than a third, similar reporting flaws were also found in other areas [26, 27]. Evidence showed that inadequate reporting of randomization and allocation concealment might exaggerate treatment effects [28]. The details about blinding in the report summary help readers understand potential bias in the results, and authors should avoid using terms such as “single” or “double” blind as such terms are not well-understood [29]. In addition, to enable readers to make rational and balanced decisions, authors should describe any important adverse (or unexpected) effects of an intervention in the abstract. If no important adverse events have occurred, the authors should state this explicitly [7, 30, 31]. Unfortunately, our study showed that none of the studies reported the harms of trials in their abstracts, which is a particularly worrying phenomenon. In RCT quality evaluation studies published in pediatrics, dermatology, and general medicine journals, it was found that the reporting rate of important adverse events or side effects in RCTs published in these journals ranged from 35 to 77% [32–34]. The studies we included were all in the nursing field, and very few used drugs and methods that could cause harm to humans. Therefore, this may be one reason why no significant adverse events or side effects were reported in the studies we included. The quality of the journals selected may also affect the results. We expect that future RCTs related to the nursing field will also pay more attention to the reporting of major adverse events or side effects.

At the same time, we found that multicenter studies had better quality summary reports, which is consistent with the results of Mbuagbaw et al. [25]. The exact reasons behind this phenomenon are yet unknown, and it can be assumed that for being from a multicenter, these studies would have more rounds of revisions between authors, thus improving the completeness of the abstract reporting. In addition, structured abstracts improve readability and facilitate a simple assessment of the information reported in the abstract. Unfortunately, even after the publication of CONSORT-A, 22 of the 152 RCTs did not use a structured format for abstracts. Of the ten journals included, except the *Journal of Family Nursing*, the remaining 9 require structured abstracts in the author guidelines. However, we found that most studies did not comply with the journal format requirements, an unstructured format leads some authors to freely organize information in abstracts, which was consistent with the results in other areas [10, 35]. This represents a need to use specific strategies to implement research-based recommendations in order to ensure a change in approach.

Another potential reason may be that some abstracts neglect to report important information due to the space constraints set by journals [36]. It is recommended that 250 to 300 words be sufficient to address all of the items in the CONSORT-A checklist [7]. Our study also showed that better reporting scores were associated with more words (> 250) in abstracts, which was indicated in other studies [20, 36, 37], although nearly half journals included in our study limit words in the abstract to less than 250, which may be due to journal preference or traditional format. The fewer word limitation for abstracts may lead to the absence of some important information. Since the abstract serves as the foundation for the initial screening of a trial, it is important to complete the reports’ abstract under recommended word limitation. It is also needed in the future to analyze the exact reasons regarding less word limitation in journals.

Our study found that journals with higher impact factors published the higher reporting quality of RCT abstracts, and similar results have been found in other areas [37–39]. Previous studies showed that journal endorsement of the CONSORT statement could improve the reporting quality of RCT abstracts [38, 40, 41], and journals with higher impact factors have higher recognition of the CONSORT statement [37]. However, it is not enough to endorse CONSORT as a full reporting guide, as some authors may ignore the reporting quality of abstracts. Thus, it is also necessary for journals to endorse the CONSORT-A guideline to assess the reporting quality of RCT abstracts [13]. Unfortunately, our study showed none of the ten journals endorsed it. Given the above, we suggest that, first of all, editors should assess their own journal’s processes for compliance with CONSORT-A, including considering whether their abstract structures and word limits hinder the possibility of compliance. Subsequently, journals should consider adopting the CONSORT-A as a prerequisite for submission. This would help the author(s) to adopt the guideline to make the abstract more reasonable and readable. Similarly, for reviewers, the completeness and efficiency of abstracts could be improved if the CONSORT-A was better disseminated [7]. The impact factor of the journals included may have changed over time. In the future, as the impact factors of selected journals improve, there may be higher requirements for the quality of RCTs.

This study has several limitations. First, the present study only included abstracts published in selected journals based on the IF of 1 year; thus, the conclusions of our study might lack applicability to journals not included in our analysis. Second, our study analyzed the completeness of reports based on the CONSORT-A checklist without considering whether the content of the abstract was accurately reflected in the full text, as this was beyond the scope of our study. Thus, further studies are needed to assess the accuracy of the full-text reports. Finally, because of the large volumes of RCTs, we only randomly chose 200 as samples with 24% pre-2009 and 76% post-2009. The results might not be representative of all nursing articles; therefore, care needs to be taken in generalizing the results.

## Conclusions

In summary, our findings suggest that the overall reporting adherence of RCT abstracts in nursing has improved significantly since the CONSORT-A publication. However, there are still gaps between the minimal items that should be reported and the actual reporting. Higher CONSORT-A scores were associated with multicenter studies, higher journal impact factors, structured abstracts, more word counts, and more recent publications. Nursing journals are the major platform for disseminating RCT research in nursing, and authors of RCT abstracts should take responsibility of reporting their research adequately. In addition, active implementation of the CONSORT-A guidelines during the submission and review period is recommended for significant improvements in the reporting of RCT abstracts.

## Data Availability

The datasets analyzed during the current study are available from the corresponding author upon reasonable request.

## Abbreviations

RCT: Randomized controlled trial
CONSORT-A: Consolidated Standards of Reporting Trials for Abstracts
MD: Mean difference
